# A novel method to follow meiotic progression in *Arabidopsis* using confocal microscopy and 5-ethynyl-2′-deoxyuridine labeling

**DOI:** 10.1186/1746-4811-10-33

**Published:** 2014-10-15

**Authors:** Patti E Stronghill, Wajma Azimi, Clare A Hasenkampf

**Affiliations:** Department of Biology, University of Toronto, 1265 Military Trail, Scarborough, Canada; Kingston General Hospital, Kingston, Canada

**Keywords:** *Arabidopsis*, Meiosis, S-phase, Prophase I, Confocal microscopy, EdU labeling, Meiocyte filament, Tapetal cells, Time course, Multi-criteria meiotic staging, DNA replication

## Abstract

**Background:**

Meiosis progression in the more recent past has been investigated using 5-bromo-2′-deoxyuridine (BrdU) uptake by S-phase meiocytes undergoing DNA replication. BrdU uptake is detected by reaction with BrdU antibody followed by epifluorescent microscopy examination of chromosome spreads and/or squashes. We here report using confocal microscopic examination of intact meiocytes in conjunction with the new thymidine analog 5-ethynyl-2′-deoxyuridine (EdU). The simplicity of the EdU detection coupled with confocal examination of anthers provides a more exact temporal description of meiotic prophase I progression in *Arabidopsis* and opens up the possibility of examining the coordination of microsporocyte development with the other tissues of the anther.

**Results:**

Using our time course protocol, we have determined the duration of wild type *Arabidopsis* leptotene to be 5 h, zygotene -6 h, pachytene -10 h and a diplotene duration of approximately 1 h. We estimate G2 duration to be approximately 7 h based on the timing of the initial appearance of EdU signal in early leptotene meiocytes. In addition we have found that DNA replication in meiocytes is not done synchronously with the associated tapetal layer of cells. The EdU labeling suggests that S-phase replication of meiocyte DNA precedes the duplication of tapetal cell DNA.

**Conclusions:**

The increased number of meiotic staging criteria that can be assessed in our confocal analysis, as compared to chromosome spreading or squashing, makes the identification of even the early and late portions of the prophase I substages attainable. This enhanced staging coupled with the ability to easily generate large data sets at hourly time points makes it possible to more exactly determine substage duration and to detect modest temporal abnormalities involving meiocyte entrance into and/or exit from leptotene, zygotene and pachytene. Confocal analysis also makes it possible to study the relationships between different cell types within the flower bud as meiosis proceeds.

**Electronic supplementary material:**

The online version of this article (doi:10.1186/1746-4811-10-33) contains supplementary material, which is available to authorized users.

## Background

S-phase is the portion of both the mitotic and meiotic cell cycle when DNA replicates. Van’t Hof [1], used tritiated thymidine labeling of meristematic root tips to investigate the duration of the mitotic cycle. He reported that mitotic cycle duration was varied amongst plants and that plants with relatively long mitotic cycles also possessed longer S-phases. Some years later tritiated thymidine labeling of S-phase meiocytes during DNA replication was used to investigate substage duration in plant pollen mother cells from wheat (*Triticum aestivum*), and rye (*Secale cereale*) [2]. Systematic studies have revealed that the S-phase that precedes meiosis is longer than that preceding mitosis for a given species [3]. Since then tritiated thymidine labeling has been replaced by the introduction of thymidine-based analogs into the replicating DNA of S-phase meiocytes. The first nucleoside analog to be used was 5-bromo-2′-deoxyuridine; this labeling was followed by the immuno-detection of BrdU and epifluorescent imaging. The timing of meiosis progression in *Arabidopsis* was first examined using chromosome spreading and BrdU uptake into S-phase male meiocytes [4–6]. The duration of prophase I substages were determined as follows; leptotene 6.0 h, zygotene/pachytene 15.3 h, diplotene to tetrad 2.7 h [5]. Subsequently, using the percentage of total cells found in the two different stages, the individual durations of zygotene and pachytene were estimated to be 4.8 h and 10 h respectively [7].

Recently a new thymidine analog 5-ethynyl-2′-deoxyuridine (EdU), has been developed by Invitrogen (California, USA). EdU labeling of DNA is detected with a fluorophore tagged azide that forms a covalent bond with the terminal acetylene group on the ethynyl component of EdU. This new technology has been extensively used in animals to study cell proliferation [8–10]. EdU incorporation has been used in conjunction with dispersed *Arabidopsis* chromosomes to determine the relative time course of the appearance of several key meiotic proteins [11]. We have developed a protocol whereby EdU label was taken up into *Arabidopsis* inflorescences by the vasculature, followed by confocal analysis of intact filaments of meiotic cells surrounded by a layer of tapetal cells. Because we used these intact filaments we were able to use features of both types of cells to define each meiotic substage and determine the duration of each substage with high precision.

## Results and discussion

### Precise estimates of all meiotic substage durations

#### Confocal microscopy permits multi-criteria staging

**Figure 1:**
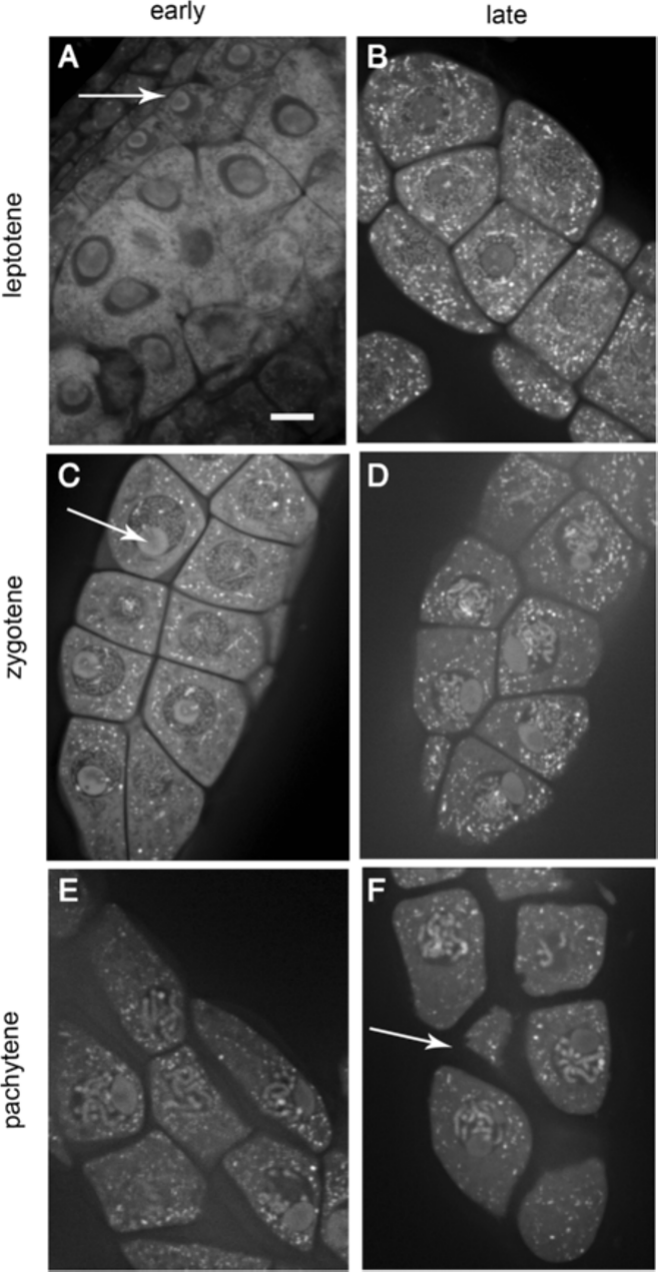
**Confocal images of meiotic filaments.** These meiotic filaments contain early **(A,C,E)** and late **(B,D,F)** leptotene, zygotene and pachytene wild type *Arabidopsis* meiocytes. These images represent a single xy slice from a multiple image z-stack. The white arrow in **(A)** indicates a mononucleate tapetal cell and in **(C)** indicates a nucleolus and in **(F)** indicates callose situated between meiocytes. Scale bar 10 um.

We stained our preparations with propidium iodide; it stains both DNA and RNA. Thus we can visualize chromosomes and the RNA-rich nucleoli. Chromosome spreads (using acid or detergent disruption of cell wall/membrane) and chromosome squashes (using physical pressure disruption of cell wall/membrane) both rely mainly on chromosome ‘thickness’ for meiotic stage identification [12]. This ‘thickness’ relates to whether homologous chromosomes are paired or not and also relates to the increasing degree of chromosome condensation that occurs as meiosis progresses [6]. Typically, spreading and to a lesser degree squashing lead to the undesirable liberation of the nucleolus [11, 13]. With our style of preparation, chromosome thickness remains an important staging tool but we also have nucleoli size, shape, and position within the meiocyte nuclei. the tapetal cell nuclei characteristics and callose deposition between meiocytes as additional diagnostic wfeatures. Our confocal meiocyte preparations either completely or partially extrude meiotic filaments from anthers thus leaving meiocytes intact and nucleoli undisturbed (Figure 1). Both the nucleoli’s shape and position within the nucleus change as meiosis proceeds; this provides valuable staging information. Partial and non-extruded filaments maintain their association with the tapetal cell layer surrounding them. Tapetal cell nuclei are mainly mononucleate in leptotene, a mixture in zygotene and binucleate in pachytene. Callose deposition between meiocytes (Figure 1) becomes thicker as prophase I progresses. Collectively these criteria make very precise meiotic staging possible. Figure 1 provides examples of meiotic filaments at both the early and late stages of leptotene, zygotene and pachytene. The criteria used to stage leptotene, zygotene, pachytene, diplotene and diakinesis meiocytes are summarized in Table 1. Using multiple staging criteria that do not rely on ‘pairing status’ is particularly important in meiotic mutant with pairing disruptions. By using multiple criteria, it should be possible to determine if the increased duration of a particular meiotic substage in a mutant meiocyte is due to premature entry, slow progression or both.

**Table 1:** **Meiotic staging criteria for wild type** ***Arabidopsis thaliana*** **prophase I substages**

Meiotic staging criteria	Sub-stages of prophase I				
	Leptotene	Zygotene	Pachytene	Diplotene	Diakinesis
**Tapetal cell nuclei**	Mononuclear	Mononuclear (early)Few binuclear (mid)Many binuclear (late)	Binuclear	Binuclear	Binuclear
**Callose wall thickness**	Very thin	Thin	Thick	Thicker	Thickest
**Nucleolus position**	Central (early)Peripheral (late)	Peripheral	Peripheral (early) Peri-central (late)	Central	Central
**Nucleolus shape**	Round	Round (early) flattened (late)	Flattened (early) round (late)	Round	Round
**Chromosome distribution in the nucleus**	Circular and takes up entire nuclear space	Crescent shape in one hemisphere of nuclear space	Roughly circular but does not take up entire nuclear space	Takes up more of nuclear space than in pachytene	Five distinct bivalents distributed in nuclear space
**Chromosome thickness**	Thin	Mixture of thin and thick	Thick	Mixture of thin and thick (early) Thick (late)	Very thick

#### Hourly time point sampling, large sample size and precise staging increases accuracy in the determination of meiotic stage duration

**Figure 2:**
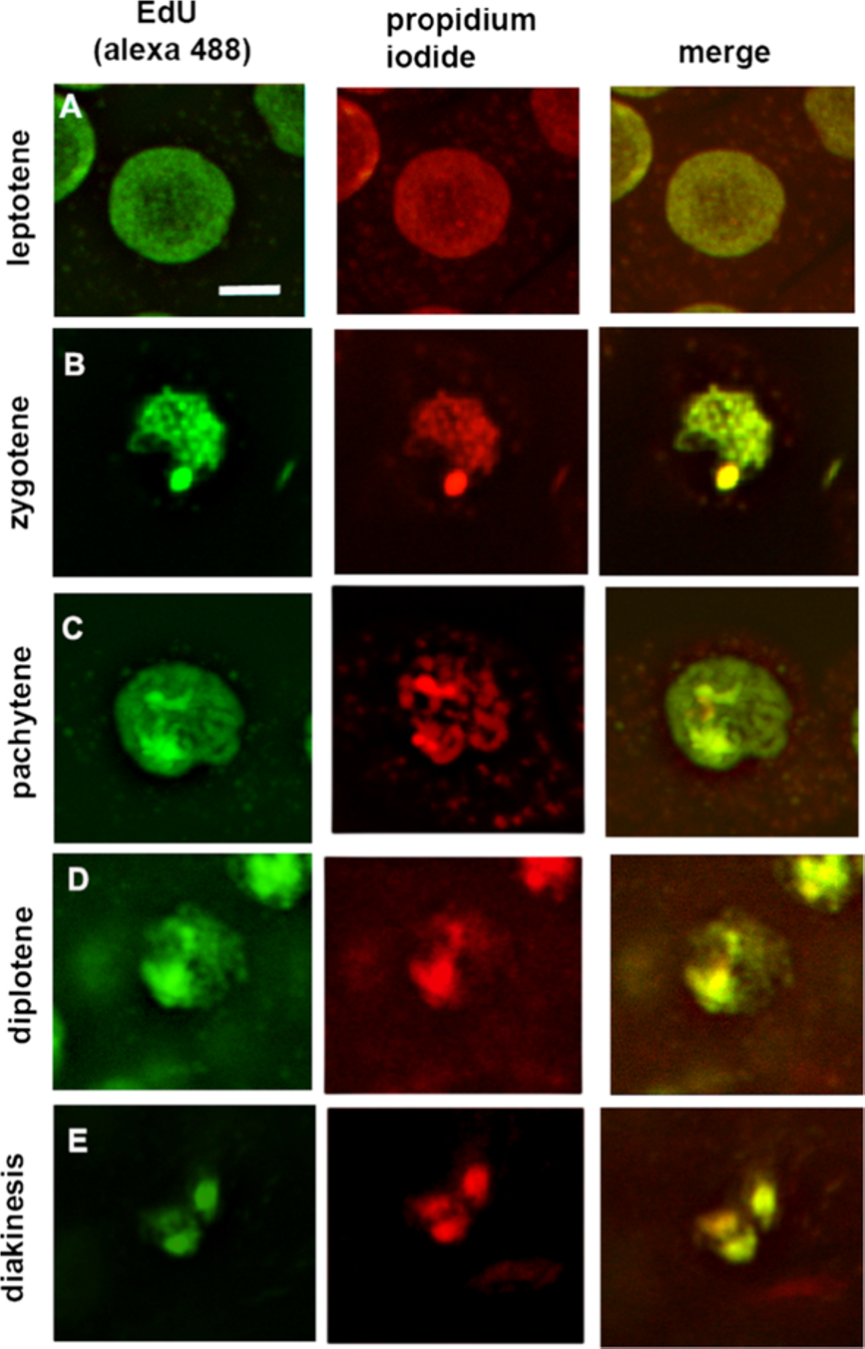
**Confocal images of EdU labeled meiocytes.** Detection of 5- ethynyl-2′-deoxyuridine(EdU) labeled. *Arabidopsis* pollen mother cells with Alexa Fluor 488 azide (green). The following prophase I substages are included: **A)** leptotene, **B)** zygotene, **C)** pachytene, **D)** diplotene, **E)** diakinesis. DNA is counterstained with propidium iodide (PI) (red). Each image is a single xy slice from a confocal z-stack. The PI stained nucleolus is not seen in these single slices but is always evident and used for staging when the entire z stack is analyzed . It should also be noted that only 3 of 5 bivalents can be seen in the diakinesis optical slice shown. Scale bar 5 um.

**Figure 3:**
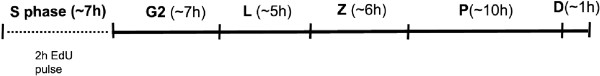
**A time course schematic of** ***Arabidopsis*** **L** ***er*** **Substage Prophase I Durations.** A meiosis prophase I time course schematic that extends from EdU pulse initiation in S-phase to diakinesis. Note: 1) that the uptake of EdU can occur at the start, middle or end of S-phase 2) S-phase duration [5] 3) rough estimate of G2 duration Leptotene (L), Zygotene (Z), Pachytene, (P), Diplotene (D).

In our time course analysis inflorescences incorporate EdU, and then are collected and fixed hourly. Subsequent preparation of meiocytes for detection of EdU labeling, using confocal microscopy, is relatively simple. This preparation produces a large number of analyzable nuclei as buds from several inflorescences, from the same time point, can be labeled together with EdU and subsequently prepared and viewed on the same slide. Examples of EdU labeling of wild type prophase I meiocytes (counterstained with propidium iodide) are shown in Figure 2. EdU, a thymidine analog stains only DNA whereas propidium iodide (PI) stains DNA, RNA and the cell wall. PI stains the nucleolus because of its RNA content. Because we are able to distinguish early from late leptotene, zygotene, and pachytene and are able to take samples for cytology at hourly time points after the addition of EdU, we can obtain precise estimations for the duration of each of the prophase I substages in wild type and an rough estimation of G2 duration (Figure 3).

EdU incorporation occurs during S-phase. The first time point (after EdU is added to anthers) in which EdU shows up in meiotic cells of a given stage is critical in determining the duration of each stage. These ‘first appearance’ time points are provided in Table 2. The first time point that EdU signal was observed in early leptotene meiocytes was 8 hours post EdU pulse initiation. This finding implies that premeiotic G2 duration is approximately 7 hours, since meiocytes that incorporated EdU into their replicating DNA, at the end of S-phase, required approximately 7 hours to progress through G2 and enter into early leptotene. The minimum time required for EdU signal to appear in early zygotene meiocytes was 13 hours after pulse initiation. The difference in timing between first appearance of EdU signal in early leptotene and early zygotene meiocytes defines leptotene duration to be 5 hours in *Arabidopsis* meiocytes (L*er* ecotype). The minimum time required for first appearance of EdU labeled early pachytene meiocytes was 19 hours post EdU pulse initiation; the difference in timing between first appearance of EdU signal in early zygotene and early pachytene meiocytes defines zygotene duration to be 6 hours. The minimum time required for first appearance of EdU labeled early diplotene meiocytes was 29 hours post EdU pulse initiation; the difference in timing between first appearance of EdU signal in early pachytene and early diplotene meiocytes defines pachytene duration to be 10 hours. Only one hour separated the first appearance of EdU signal in early diplotene and early diakinesis meiocytes. Therefore diplotene duration appears to be approximately one hour. Hourly time point-data collection is an absolute requirement for measuring the durations of diplotene, diakinesis and the remaining stages of meiosis, which are all relatively short. In previous work we determined leptotene, zygotene and pachytene durations to be 6.0, 4.8 and 10 hours respectively by an indirect method. This indirect method was based on examination of a very large number of meiotic nuclei to ascertain the percentages of meiocytes observed in each substage. These percentages were applied to the total duration of meiosis as determined by BrdU analysis to yield approximate substage durations [7]. The substage durations of leptotene (5 h), zygotene (6 h) and pachytene (10 h) presented in this paper are close to our previous values but are more precise due to the direct method of measurement. All of the data on the percentage of EdU labeled meiocyte observed, for each prophase I substage, at each time point, is given in Additional file 1: Table S1.

**Table 2:** **A summary of** ***Arabidopsis*** **L** ***er*** **prophase I substage durations**

Meiotic stage	Time from EdU pulse initiation to first labeling (h)	Stage duration (h)
**Leptotene**	8	5
**Zygotene**	13	6
**Pachytene**	19	10
**Diplotene**	29	1
**Diakinesis**	30	

From this work, we have found that EdU labeling is well suited for confocal microscopy analysis of whole meiotic filaments as the fluorophore tagged azide penetrates the tissue, all the way into meiocytes more easily than the antibody required for BrdU detection. The EdU signal obtained was easily observed in *Arabidopsis* meiocytes (Figure 2). The preparation of meiocytes for confocal examination is simpler than for chromosome spread preparations, making the analysis of hourly sampled EdU labeled meiocytes feasible. In addition the number of analyzable meiocytes for each time point is much greater. Consequently the data set from our confocal microscopy- based, time course analysis is significantly larger than for other methods and allows us to more precisely study meiotic progression.

### Relationship between tapetal and meiocyte DNA replication

The primary purpose of our study was to develop a precise, simplified method for labeling DNA during S-phase for the purpose of determining the duration of subsequent meiotic stages in normal and aberrant situations. However since confocal microscopy can be used to create optical sectioning through multiple cell layers, we decided to explore the use of this feature with EdU labeling to address questions relating to coordination of events between cell layers of the anthers. We therefore examined EdU incorporation in the tapetum, the cell layer that surrounds meiotic cells of the anther. The tapetal cells are known to undergo a relatively synchronous mitosis at the transition between zygotene and pachytene; we did a preliminary look at when tapetal cells replicate their DNA.

**Figure 4:**
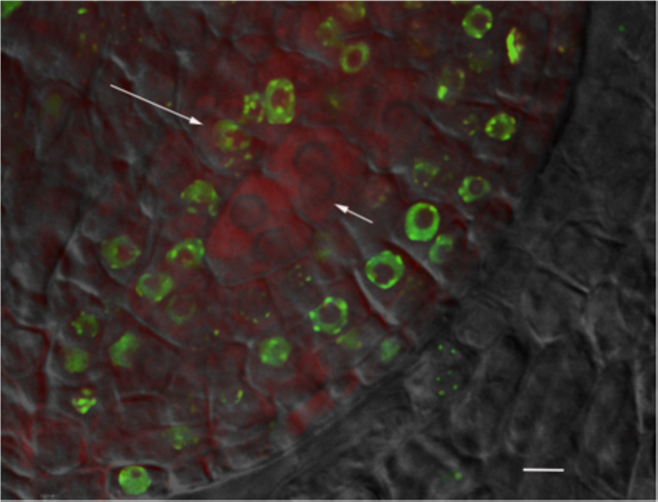
**Edu labeling of a meiotic filament.** A representative image of a meiotic filament comprised of non-EdU labeled, leptotene meiocytes (short arrow) surrounded by a layer of EdU labeled tapetal cell nuclei (long arrow). EdU labeling (green) and PI counterstain (red) This image is a single xy slice from a confocal z-stack. Scale bar 10 um.

To study tapetal cells we examined those of our cytological preparations that had only partially extruded the filaments of meiotic cells from the anthers and were associated with the surrounding tapetal layer. EdU labeling pattern was observed in 50 partially and non-extruded meiotic filaments from three different time course experiments. Our observations are summarized in Table 3. Four patterns are theoretically possible based on whether or not the tapetal and/or meiotic cells were labeled. Pattern 1 - Meiotic and tapetal cells are not labeled. This pattern would be generated in meiotic filaments that did not incorporate EdU in the time frame of our experiment, producing unlabeled meiotic and tapetal cells. This pattern was observed. Pattern 2 - Meiotic and tapetal cells are both labeled. This pattern could be generated in filaments that contained meiotic cells undergoing S-phase when the EdU was present as well as tapetal cells undergoing S-phase at the same time or after the meiotic cells. This pattern was observed. Pattern 3- Meiotic cells are labeled, but tapetal cells are not labeled. This pattern could be generated in filaments if meiotic cells underwent S-phase when the EdU was present, but tapetal cell DNA replication only occurred before EdU addition. This pattern was not observed. Pattern 4- Meiotic cells are not labeled but tapetal cells are labeled. This pattern could be generated if the meiotic cells had already completed S-phase before EdU was present but tapetal cell DNA replication had not yet occurred. We did see this pattern and in fact when we saw this pattern, all of the cells in the tapetal layer were labeled with EdU (Figure 4). The existence of this last pattern demonstrates that tapetal cells undergo DNA replication after the meiotic cells have completed their last DNA replication.

**Table 3:** **Combinations of EdU labeling patterns observed in partially extruded anthers**

EdU labeling patterns observed in meiotic filaments (n = 50)			
Pattern #	Meiocytes	Tapetal cells	# of filaments examined
**1**	Not labeled	Not labeled	16
**2**	Labeled	Labeled	14
**3**	Labeled	Lot labeled	0
**4**	Not labeled	Labeled	20

Furthermore we noticed that for the meiotic cells of one anther chamber, either none of the cells were labeled, or all were labeled. This is not surprising since the meiotic cells are synchronized from pre-meiotic S-phase onward [7]. The tapetal cell layer also had this same ‘all or none’ labeling pattern. Therefore either the tapetal cells all undergo synchronous DNA replication after the meiotic cells have completed S-phase, or they are cycling fast enough such that all tapetal cells have undergone an additional unsynchronized replication in the time period of EdU exposure. Future experiments, designed specifically to address this question will be done.

## Conclusions

Confocal examination of EdU labeled meiocytes allows for a more precise analysis of meiotic substage duration in wild type and presents the opportunity to study possible temporal effects on meiotic progression in meiotic mutants. This claim is based on the increased precision of meiotic staging, hourly sampling and the much larger sampling size that our confocal method allows. Our confocal time course method promises to be a powerful tool that can be used, in future, to identify and pinpoint even subtle abnormalities in stage duration and meiotic progression. Furthermore coordination of DNA replication in the various anther cell types can be evaluated. The method also could be easily adapted to the study of meiosis in megasporocytes within plant gynoecium. Entire intact EdU labeled anthers can be analyzed with confocal microscopy to allow for studies of coordination of meiosis within the development of the other tissues of anthers.

## Method

### Plant material

Seeds of *A. thaliana* cv. Landsberg *erecta* (L*er*) were obtained from the *Arabidopsis* Biological Resource Center (http://www.arabidopsis.org/abrc). Plants were grown under long day conditions (16 h light/8 h dark); the day/night temperatures were 22°C/19°C. Light intensity was ~170 umoles/m^2^/s. Plants were ready to be used in the time course experiment when the stems of primary inflorescences were approximately 10 cm long.

### EdU S-phase labeling

Stems of young inflorescences (1–3 opened flowers) were quickly cut under tap water to a length of approximately 9 cm. The cut ends were immediately submerged in a small quantity of 10 mM EdU labeling solution from a Click-IT assay kit (Invitrogen, California, USA). These inflorescences were placed under grow lights at 21°C for 2 h (light intensity ~170 um/m^2^/s) to allow the EdU to reach the buds via their vasculature and subsequently to be incorporated into the DNA of S-phase cells. After the 2 h pulse the EdU was exchanged with tap water several times to stop addition of EdU to the nucleotide pool. After these rinses inflorescences in tap water were returned to the grow lights and at hourly intervals two inflorescences were sampled. Negative controls consisted of inflorescences prepared exactly as experimental ones except stems were placed in water only (no EdU).

### Inflorescence fixation

For each time-point the inflorescences were processed as follows. Larger buds (beyond meiosis) were removed from the inflorescence and a couple of small holes were made in the remaining attached buds (to facilitate formaldehyde uptake), taking care not to damage the anthers within. The inflorescences were then placed in 3.7% formaldehyde in meiocyte buffer A [15 mM pipes-NaOH (pH 6.8), 80 mM KCl, 20 mM NaCl, 0.5 mM EGTA, 2 mM EDTA, 0.15 mM spermine tetra-HCl, 0.05 mM spermidine, 1 mM dithiothreitol, 0.32 M sorbitol] (pH =8.2). Vacuum infiltration was done (70 psi) at room temperature for 30 min to assist fixative penetration. After fixation the inflorescences were rinsed 3 × 10 min in meiocyte buffer A and then stored in meiocyte buffer A at 4°C until used, typically within 24 hours.

### EdU detection

Inflorescences from the first time point were brought to room temperature and washed twice with 3% Bovine Serum Albumin (BSA) in Phosphate Buffered Saline (PBS) (pH =7.4) and then transferred to a depression slide containing the same solution. The sepals and petals were removed from 0.3-0.5 mm buds leaving stamens and pistils intact. These buds with the two outer whorls removed, were then transferred to an epitube containing 3% BSA in PBS. This step was repeated for the inflorescences from all the remaining time points. After brief centrifugation the 3% BSA in PBS was exchanged twice with 0.5% triton X in PBS and allowed to incubate at room temperature for 60 min. Next the intact pistil-stamens were rinsed twice in 3% BSA in PBS, then placed in EdU Click-It colour reaction cocktail as per manufacturer’s instructions (Invitrogen) for one hour at room temperature in the dark. The intact pistil-stamens were then rinsed twice in 1 ml BSA in PBS and kept in the dark until used for cytology.

### Cytological preparation

The intact pistil-stamens for the first time point, were removed from 3% BSA in PBS. Using a dissecting microscope, anthers were removed from the stamens, scored with a scalpel at their midpoint and transferred to approximately 15 ul of propidium iodide in Vectashield (Vector Laboratories, Burlington, Canada) that had been placed in the center of a clean slide. A 22 × 22 mm No. 1 coverslip was placed over the sample area and a slight pressure applied and the edges of the coverslip were sealed with nail polish. This procedure was repeated for the pistil-stamens for all the remaining time points.

### Microscopic analysis

Slides were observed using a Quorum Wave-FX spinning disk confocal microscope and a Leica 63X oil immersion Plan-apo objective (NA 1.4). The solid state lasers used were 491 nm and 561 nm. The emission filters used were bandpass 525/50 nm and bandpass 620/60 nm respectively. The CCD camera used to capture images was a Hamamatsu Orca R2. The software used for image capture and analysis were Metamorph version 7.7.9.0 and Velocity version 6.1.1. Photoshop CS2 was used to label images and create figure montages.

### Meiotic staging

Our confocal microscopy sample preparation method extruded meiotic filaments (a structure comprised of ~30 meiocytes) from anther locules. The filaments were either completely or partially extruded. Partially extruded filaments had the advantage of preserving some tapetal–meiocyte associations and in these instances tapetal cells were also used in the staging process. We have taken advantage of the attributes of both the meiocytes and the tapetal cells in the staging of meiotic cells [14, 15]. The amount of callose deposition between meiocytes and the nuclear morphology of tapetal cells also served as criteria in meiotic staging. As well intact meiocytes retained the nucleolus (propidium iodide labels the nucleolus), whose shape and position within the nucleus changes as meiosis proceeds, served as an additional valuable staging tool. Collectively this information made it possible to more precisely identify meiotic stages. Furthermore we used our precise multi-criteria staging to identify the early and late portions of leptotene, zygotene and pachytene substages. The criteria we used for precise meiotic staging are outlined in Table 1.

## Electronic supplementary material

Additional file 1: Table S1: Time course data for all meiotic filaments examined. (DOC 69 KB)

## Abbreviations

BrdU: 5-bromo-2′-deoxyuridine
EdU: 5-ethynyl-2′-deoxyuridine
PMC: Pollen mother cell
CCD: Charge coupled device.

## References

[1] Van’t Hof J (1965). Relationships between mitotic cycle duration, S period duration and the average rate of DNA synthesis in the root meristem cells of several plants. Exp Cell Res.

[2] Bennet MD, Chapman V, Riley R (1971). The duration of meiosis in pollen mother cells of wheat, rye and *Triticale*. Proc Roy Soc Lond B.

[3] Strich R (2004). Meiotic DNA Replication. Curr Topics in Dev Biol.

[4] Armstrong S, Franklin FCH, Jones GH (2001). Nucleolus-associated telomere clustering and pairing precede meiotic chromosome synapsis in *Arabidopsis thaliana*. J Cell Sci.

[5] Armstrong SJ, Franklin FCH, Jones GH (2003). A meiotic time course for *Arabidopsis thaliana*. Sex Plant Repro.

[6] Armstrong SJ, Jones GH (2003). Meiotic cytology and chromosome behaviour in wild type *Arabidopsis thaliana*. J Exp Biol.

[7] Stronghill P, Hasenkampf CA (2007). Analysis of Substage Associations in Prophase I of Meiosis in Floral Buds of Wild Type *Arabidopsis thaliana (Brassicaceae*). Amer J Bot.

[8] Warren M, Puskarcyzk K, Chapman SC (2009). Chick embryo proliferation studies using EdU labeling. Dev Dynamics.

[9] Yu Y, Arora A, Roifman CM, Grunebaum E (2009). EdU incorporation is an alternative non-radioactive assay to [(3)H]thymidine uptake for in vitro measurement of mice T-cell proliferations. J Immunol Methods.

[10] Mead TJ, Lefebvre V (2014). Proliferation Assays (BrdU and EdU) on Skeletal Tissue Sections. Focus.

[11] Armstrong SJ (2013). A time course for the analysis of meiotic progression in *Arabidopsis thaliana*. Methods in Mol Biol.

[12] Ross KJ, Fransz P, Jones GH (1996). A light microscopic atlas of meiosis in *Arabidopsis thaliana*. Chromosome Res.

[13] Sanchez- Moran E, Santos J-L, Jones GH, Franklin FCH (2007). ASY1 mediates AtDMC1-dependent interhomolog recombination during meiosis in *Arabidopsis*. Genes & Dev.

[14] Owen HA, Makaroff CA (1995). Ultrastructure of microsporogenesis and microgametogenesis in *Arabidopsis thaliana*. Protoplasma.

[15] Pathan N, Stronghill P, Hasenkampf C (2013). Transmission electron microscopy and serial reconstructions reveal novel meiotic phenotypes for the *ahp2* Mutant of *Arabidopsis thaliana*. Genome.

