# Supplementary material for: A novel method to follow meiotic progression in Arabidopsis using confocal microscopy and 5-ethynyl-2′-deoxyuridine labeling
Source: Plant Methods. 2014 Oct 15;10:33. doi: 10.1186/1746-4811-10-33 (PMC4203904; doi:10.1186/1746-4811-10-33)
Supplement: Supplementary file 1 — Additional file 1: Table S1: Time course data for all meiotic filaments examined. (DOC 69 KB) [file 13007_2014_300_MOESM1_ESM.doc]

**Additional file 1: table S1 Time course data for all meiotic filaments examined.**

| **Time point** | **Leptotene PMC** | **Zygotene PMC** | **Pachytene PMC** | **Diplotene PMC** | **Diakenesis PMC** |
| --- | --- | --- | --- | --- | --- |
| **(hours post** | **# with sig/#total PMC (%)** | **#with sig/#total PMC (%)** | **# with sig/#total PMC (%)** | **# with sig/#total PMC (%)** | **# with sig/#total PMC (%)** |
| **pulse initiation)** |  |  |  |  |  |
|  |  |  |  |  |  |
|  |  |  |  |  |  |
| 6 | 0/330 (0) | 0/120 (0) | 0/120 (0) | ~ | 0/120 (0) |
| 7 | 0/90 (0) | 0/180 (0) | 0/240 (0) | 0/60 (0) | ~ |
| 8 | **180/180 (100)** | 0/30 (0) | ~ | ~ | ~ |
| 9 | ~ | 0/60 (0) | ~ | ~ | 0/60 (0) |
| 10 | 60/120 (50) | 0/60 (0) | 0/150 | ~ | ~ |
| 11 | 60/60 (100) | ~ | 0120 (0) | 0/60 (0) | 0/30 (0) |
| 12 | 90/330 (27) | 0/150 (0) | 0/120 (0) | 0/120 (0) | 0/60 (0) |
| 13 | 30/30(100) | **240/480 (50)** | 0/60 (0) | 0/60 (0) | 0/60 (0) |
| 14 | 30/30 (100) | 100/270 (37) | 0/160 (0) | ~ | ~ |
| 15 | 60/60 (100) | 240/240 (100) | 0/60 (0) | ~ | ~ |
| 16 | 7/150 (100) | ~ | 0/150 (0) | ~ | 0/67 (0) |
| 17 | 115/150 (77) | 300/330 (91) | 0/65 (0) | 0/30 (0) | 0/120 (0) |
| 18 | 22/210 (18) | ~ | 0/297 (0) | ~ | ~ |
| 19 | 60/120 (50) | 100/120 (83) | **60/120 (50)** | 0/30 (0) | 0/150 (0) |
| 20 | 24/150 (16) | 180/180 (100) | 30/30 (100) | 0/30 (0) | ~ |
| 21 | 12/60 (20) | 120/120 (100) | 435/480 (91) | ~ | ~ |
| 22 | 10/120 (8) | 195/240 (81) | 60/240 (25) | 0/60 (0) | ~ |
| 23 | 75/90 (75) | 240/240 (100) | 150/210 (71) | 0/30 (0) | 0/150 (0) |
| 24 | 48/630 (8) | 30/30 (100) | ~ | ~ | ~ |
| 25 | 0/90 (0) | ~ | 30/150 (20) | ~ | 0/120 (0) |
| 26 | 0/60(0) | ~ | 210/330 (64) | 0/60(0) | 0/30 (0) |
| 27 | 90/210 (43) | 120/120 (100) | 60/180 (33) | ~ | 0/60 (0) |
| 28 | ~ | 90/150 (60) | 240/240 (100) | 0/90 (0) | ~ |
| 29 | 45/90 (50) | 17/90 (19) | ~ | **30/60 (50)** | 0/90 (0) |
| 30 | 0/300 (0) | 252/300 (84) | ~ | 30/30 (100) | **20/30 (67)** |
|  |  |  |  |  |  |
|  |  |  |  |  |  |
|  |  |  |  |  |  |
|  |  |  |  |  |  |

Bolded data indicates the time point at which EdU signal was first observed for that particular prophase I substage
